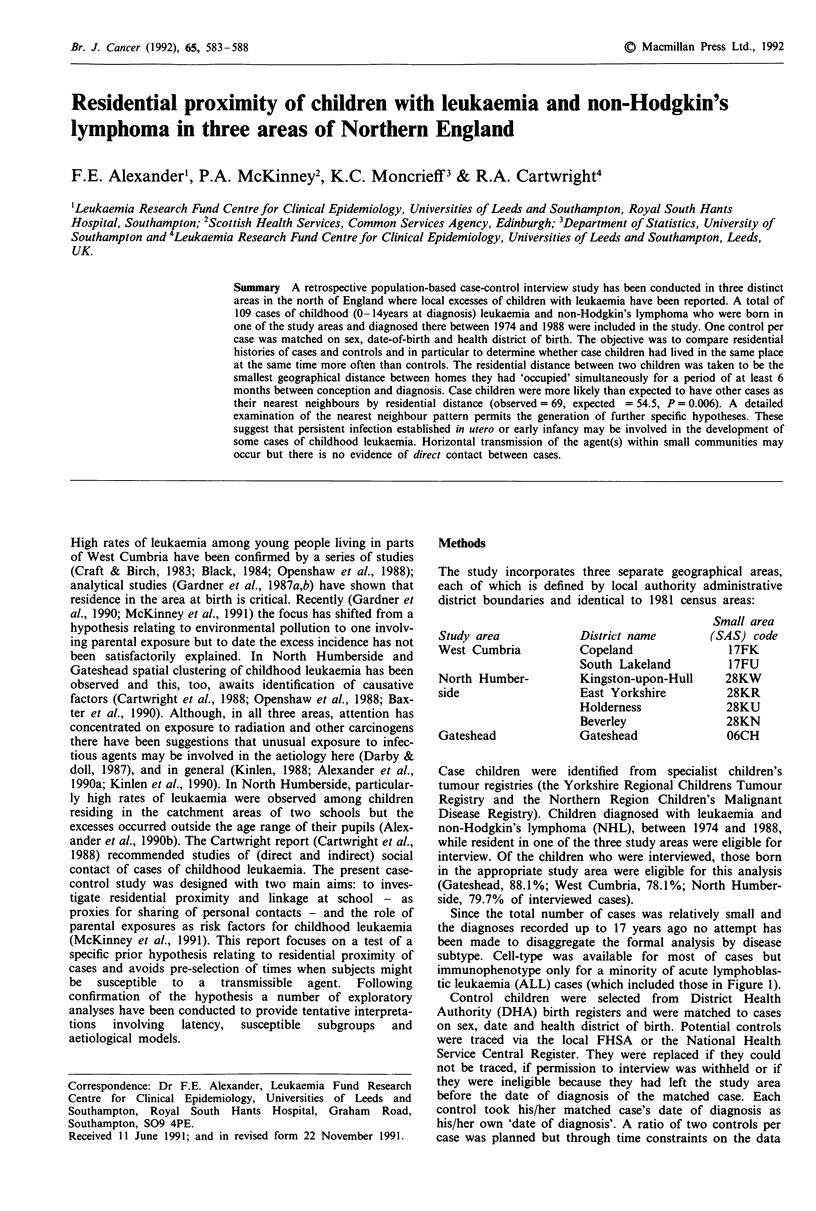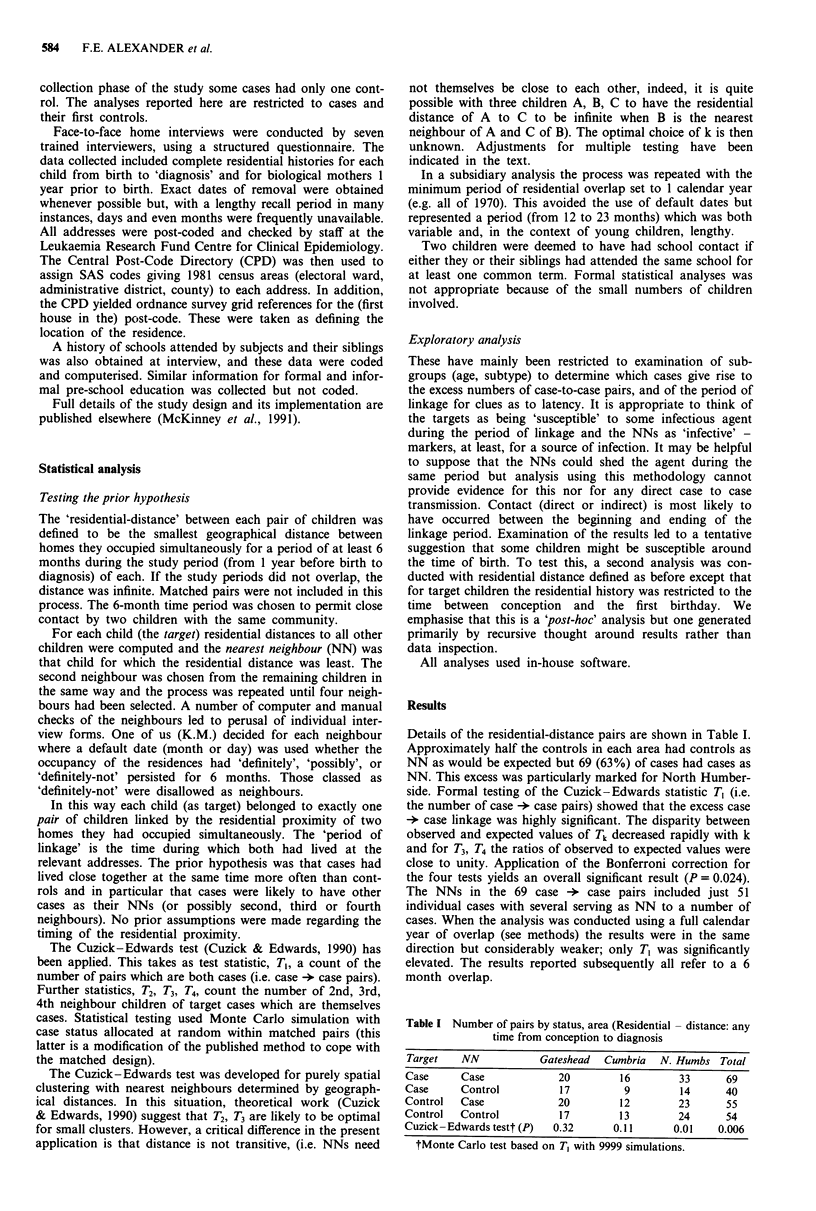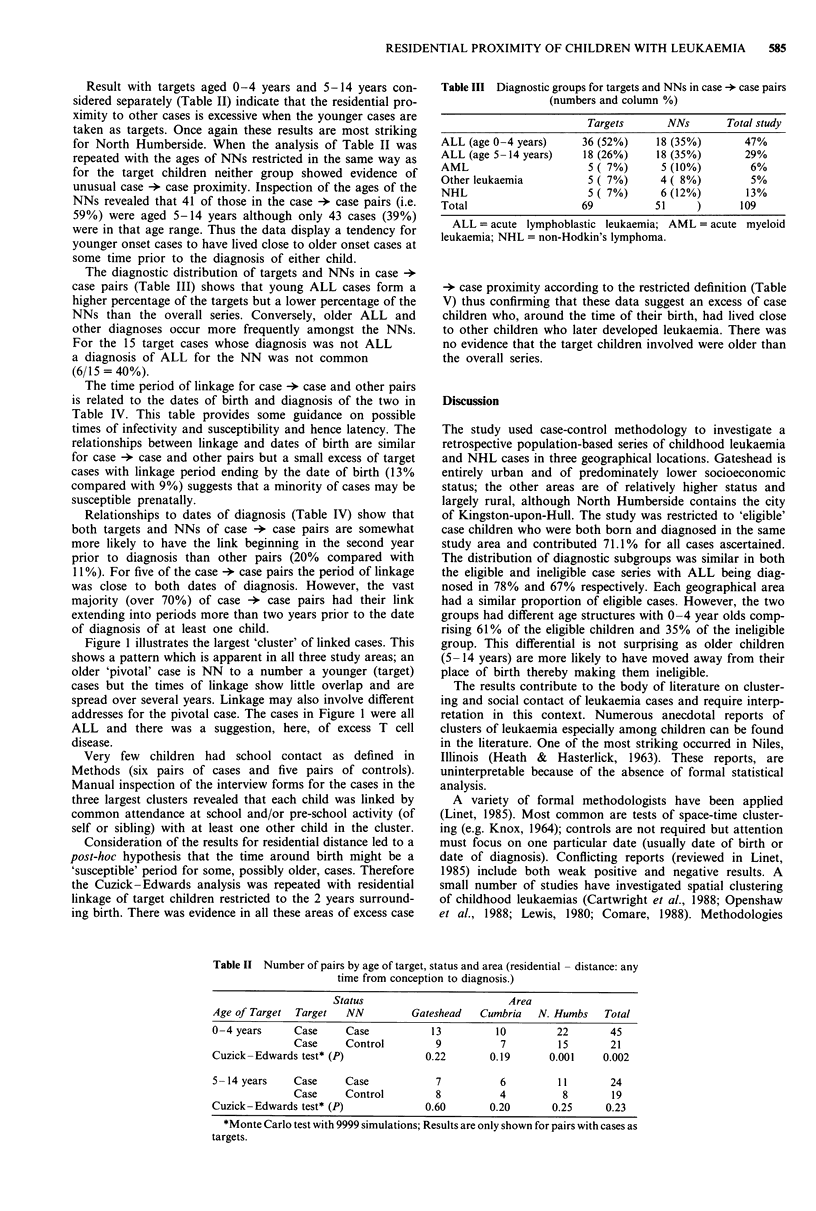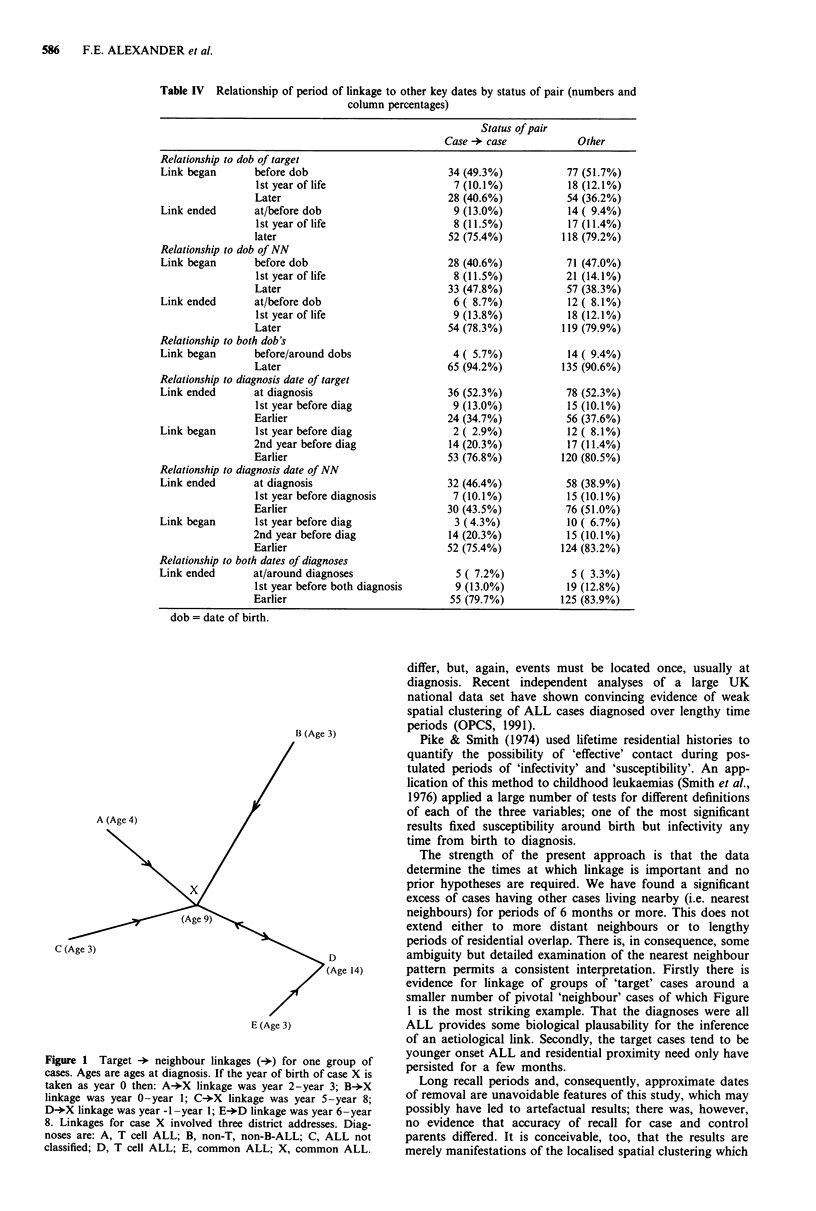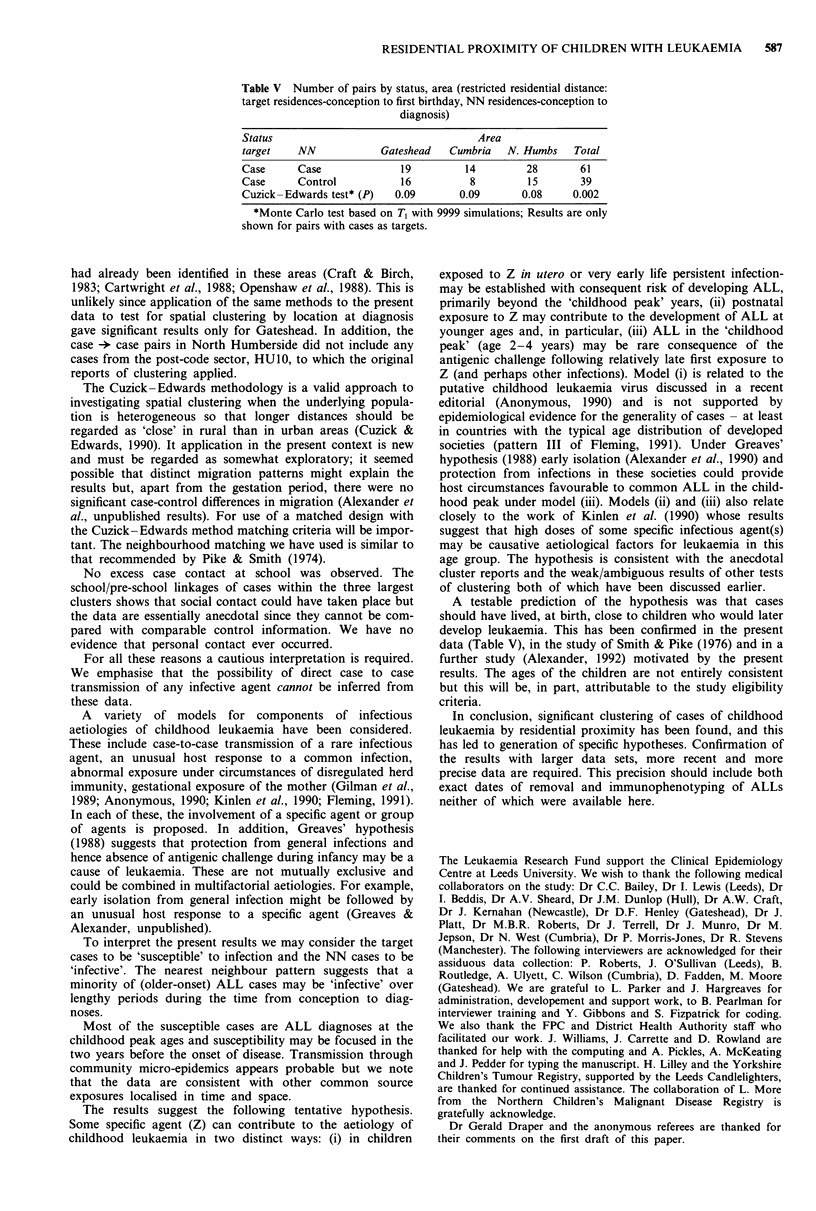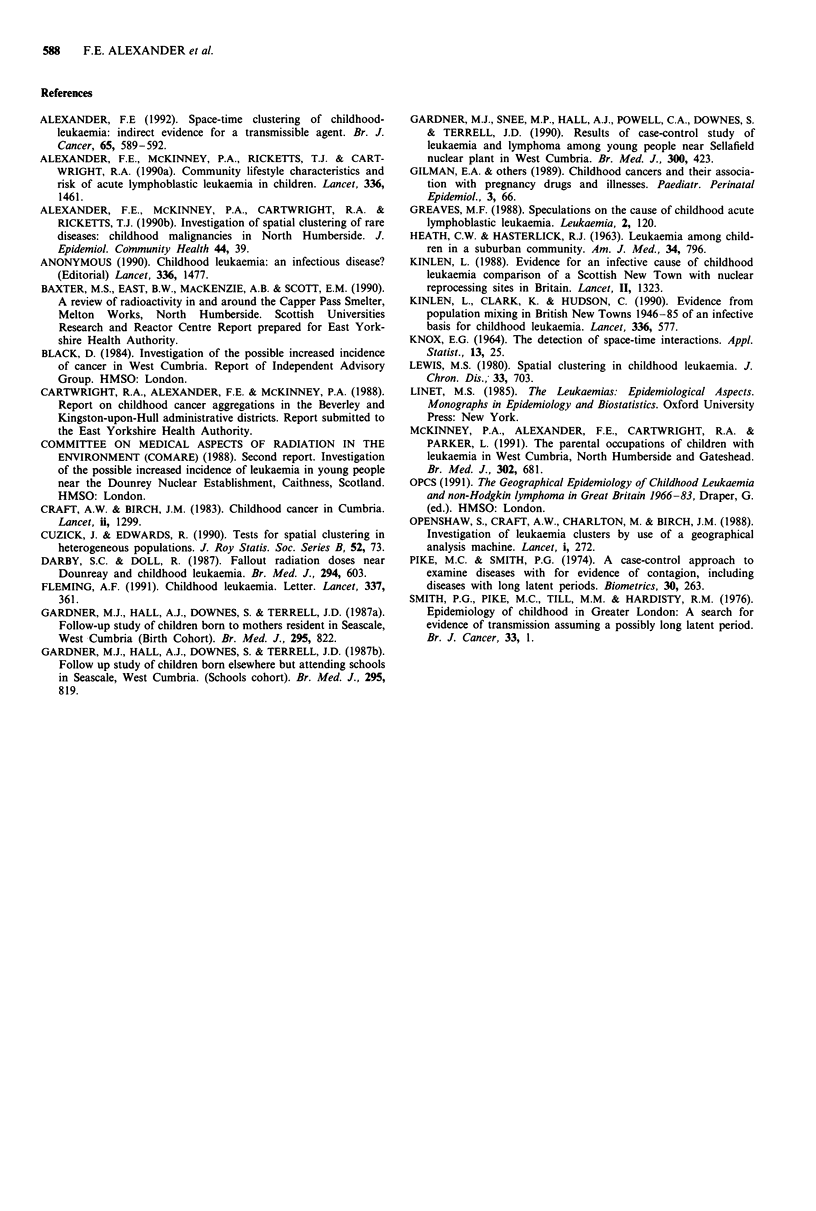# Residential proximity of children with leukaemia and non-Hodgkin's lymphoma in three areas of northern England.

**DOI:** 10.1038/bjc.1992.118

**Published:** 1992-04

**Authors:** F. E. Alexander, P. A. McKinney, K. C. Moncrieff, R. A. Cartwright

**Affiliations:** Leukaemia Research Fund Centre for Clinical Epidemiology, University of Leeds, U.K.

## Abstract

A retrospective population-based case-control interview study has been conducted in three distinct areas in the north of England where local excesses of children with leukaemia have been reported. A total of 109 cases of childhood (0-14 years at diagnosis) leukaemia and non-Hodgkin's lymphoma who were born in one of the study areas and diagnosed there between 1974 and 1988 were included in the study. One control per case was matched on sex, date-of-birth and health district of birth. The objective was to compare residential histories of cases and controls and in particular to determine whether case children had lived in the same place at the same time more often than controls. The residential distance between two children was taken to be the smallest geographical distance between homes they had 'occupied' simultaneously for a period of at least 6 months between conception and diagnosis. Case children were more likely than expected to have other cases as their nearest neighbours by residential distance (observed = 69, expected = 54.5, P = 0.006). A detailed examination of the nearest neighbour pattern permits the generation of further specific hypotheses. These suggest that persistent infection established in utero or early infancy may be involved in the development of some cases of childhood leukaemia. Horizontal transmission of the agent(s) within small communities may occur but there is no evidence of direct contact between cases.


Br. J. Cancer (1992), 65, 583-588

© Macmillan Press Ltd., 1992

### Residential proximity of children with leukaemia and non-Hodgkin's lymphoma in three areas of Northern England

F.E. Alexander[1], P.A. McKinney[2], K.C. Moncrieff[3] & R.A. Cartwright[4]

[1]*Leukaemia Research Fund Centre for Clinical Epidemiology, Universities of Leeds and Southampton, Royal South Hants Hospital, Southampton;* [2]*Scottish Health Services, Common Services Agency, Edinburgh;* [3]*Department of Statistics, University of Southampton and* [4]*Leukaemia Research Fund Centre for Clinical Epidemiology, Universities of Leeds and Southampton, Leeds, UK.*

**Summary** A retrospective population-based case-control interview study has been conducted in three distinct areas in the north of England where local excesses of children with leukaemia have been reported. A total of 109 cases of childhood (0-14years at diagnosis) leukaemia and non-Hodgkin's lymphoma who were born in one of the study areas and diagnosed there between 1974 and 1988 were included in the study. One control per case was matched on sex, date-of-birth and health district of birth. The objective was to compare residential histories of cases and controls and in particular to determine whether case children had lived in the same place at the same time more often than controls. The residential distance between two children was taken to be the smallest geographical distance between homes they had 'occupied' simultaneously for a period of at least 6 months between conception and diagnosis. Case children were more likely than expected to have other cases as their nearest neighbours by residential distance (observed = 69, expected = 54.5, $P = 0.006$). A detailed examination of the nearest neighbour pattern permits the generation of further specific hypotheses. These suggest that persistent infection established *in utero* or early infancy may be involved in the development of some cases of childhood leukaemia. Horizontal transmission of the agent(s) within small communities may occur but there is no evidence of *direct* contact between cases.

High rates of leukaemia among young people living in parts of West Cumbria have been confirmed by a series of studies (Craft & Birch, 1983; Black, 1984; Openshaw *et al.*, 1988); analytical studies (Gardner *et al.*, 1987*a,b*) have shown that residence in the area at birth is critical. Recently (Gardner *et al.*, 1990; McKinney *et al.*, 1991) the focus has shifted from a hypothesis relating to environmental pollution to one involving parental exposure but to date the excess incidence has not been satisfactorily explained. In North Humberside and Gateshead spatial clustering of childhood leukaemia has been observed and this, too, awaits identification of causative factors (Cartwright *et al.*, 1988; Openshaw *et al.*, 1988; Baxter *et al.*, 1990). Although, in all three areas, attention has concentrated on exposure to radiation and other carcinogens there have been suggestions that unusual exposure to infectious agents may be involved in the aetiology here (Darby & doll, 1987), and in general (Kinlen, 1988; Alexander *et al.*, 1990a; Kinlen *et al.*, 1990). In North Humberside, particularly high rates of leukaemia were observed among children residing in the catchment areas of two schools but the excesses occurred outside the age range of their pupils (Alexander *et al.*, 1990b). The Cartwright report (Cartwright *et al.*, 1988) recommended studies of (direct and indirect) social contact of cases of childhood leukaemia. The present case-control study was designed with two main aims: to investigate residential proximity and linkage at school - as proxies for sharing of personal contacts - and the role of parental exposures as risk factors for childhood leukaemia (McKinney *et al.*, 1991). This report focuses on a test of a specific prior hypothesis relating to residential proximity of cases and avoids pre-selection of times when subjects might be susceptible to a transmissible agent. Following confirmation of the hypothesis a number of exploratory analyses have been conducted to provide tentative interpretations involving latency, susceptible subgroups and aetiological models.

Correspondence: Dr F.E. Alexander, Leukaemia Fund Research Centre for Clinical Epidemiology, Universities of Leeds and Southampton, Royal South Hants Hospital, Graham Road, Southampton, SO9 4PE.
Received 11 June 1991; and in revised form 22 November 1991.

### Methods

The study incorporates three separate geographical areas, each of which is defined by local authority administrative district boundaries and identical to 1981 census areas:

| *Study area* | *District name* | *Small area (SAS) code* |
|---|---|---|
| West Cumbria | Copeland | 17FK |
| | South Lakeland | 17FU |
| North Humberside | Kingston-upon-Hull | 28KW |
| | East Yorkshire | 28KR |
| | Holderness | 28KU |
| | Beverley | 28KN |
| Gateshead | Gateshead | 06CH |

Case children were identified from specialist children's tumour registries (the Yorkshire Regional Childrens Tumour Registry and the Northern Region Children's Malignant Disease Registry). Children diagnosed with leukaemia and non-Hodgkin's lymphoma (NHL), between 1974 and 1988, while resident in one of the three study areas were eligible for interview. Of the children who were interviewed, those born in the appropriate study area were eligible for this analysis (Gateshead, 88.1%; West Cumbria, 78.1%; North Humberside, 79.7% of interviewed cases).

Since the total number of cases was relatively small and the diagnoses recorded up to 17 years ago no attempt has been made to disaggregate the formal analysis by disease subtype. Cell-type was available for most of cases but immunophenotype only for a minority of acute lymphoblastic leukaemia (ALL) cases (which included those in Figure 1).

Control children were selected from District Health Authority (DHA) birth registers and were matched to cases on sex, date and health district of birth. Potential controls were traced via the local FHSA or the National Health Service Central Register. They were replaced if they could not be traced, if permission to interview was withheld or if they were ineligible because they had left the study area before the date of diagnosis of the matched case. Each control took his/her matched case's date of diagnosis as his/her own 'date of diagnosis'. A ratio of two controls per case was planned but through time constraints on the data collection phase of the study some cases had only one control. The analyses reported here are restricted to cases and their first controls.

Face-to-face home interviews were conducted by seven trained interviewers, using a structured questionnaire. The data collected included complete residential histories for each child from birth to 'diagnosis' and for biological mothers 1 year prior to birth. Exact dates of removal were obtained whenever possible but, with a lengthy recall period in many instances, days and even months were frequently unavailable. All addresses were post-coded and checked by staff at the Leukaemia Research Fund Centre for Clinical Epidemiology. The Central Post-Code Directory (CPD) was then used to assign SAS codes giving 1981 census areas (electoral ward, administrative district, county) to each address. In addition, the CPD yielded ordnance survey grid references for the (first house in the) post-code. These were taken as defining the location of the residence.

A history of schools attended by subjects and their siblings was also obtained at interview, and these data were coded and computerised. Similar information for formal and informal pre-school education was collected but not coded.

Full details of the study design and its implementation are published elsewhere (McKinney *et al.*, 1991).

### Statistical analysis

### *Testing the prior hypothesis*

The 'residential-distance' between each pair of children was defined to be the smallest geographical distance between homes they occupied simultaneously for a period of at least 6 months during the study period (from 1 year before birth to diagnosis) of each. If the study periods did not overlap, the distance was infinite. Matched pairs were not included in this process. The 6-month time period was chosen to permit close contact by two children with the same community.

For each child (the *target*) residential distances to all other children were computed and the *nearest neighbour* (NN) was that child for which the residential distance was least. The second neighbour was chosen from the remaining children in the same way and the process was repeated until four neighbours had been selected. A number of computer and manual checks of the neighbours led to perusal of individual interview forms. One of us (K.M.) decided for each neighbour where a default date (month or day) was used whether the occupancy of the residences had 'definitely', 'possibly', or 'definitely-not' persisted for 6 months. Those classed as 'definitely-not' were disallowed as neighbours.

In this way each child (as target) belonged to exactly one *pair* of children linked by the residential proximity of two homes they had occupied simultaneously. The 'period of linkage' is the time during which both had lived at the relevant addresses. The prior hypothesis was that cases had lived close together at the same time more often than controls and in particular that cases were likely to have other cases as their NNs (or possibly second, third or fourth neighbours). No prior assumptions were made regarding the timing of the residential proximity.

The Cuzick–Edwards test (Cuzick & Edwards, 1990) has been applied. This takes as test statistic, $T_1$, a count of the number of pairs which are both cases (i.e. case → case pairs). Further statistics, $T_2$, $T_3$, $T_4$, count the number of 2nd, 3rd, 4th neighbour children of target cases which are themselves cases. Statistical testing used Monte Carlo simulation with case status allocated at random within matched pairs (this latter is a modification of the published method to cope with the matched design).

The Cuzick–Edwards test was developed for purely spatial clustering with nearest neighbours determined by geographical distances. In this situation, theoretical work (Cuzick & Edwards, 1990) suggest that $T_2$, $T_3$ are likely to be optimal for small clusters. However, a critical difference in the present application is that distance is not transitive, (i.e. NNs need not themselves be close to each other, indeed, it is quite possible with three children A, B, C to have the residential distance of A to C to be infinite when B is the nearest neighbour of A and C of B). The optimal choice of k is then unknown. Adjustments for multiple testing have been indicated in the text.

In a subsidiary analysis the process was repeated with the minimum period of residential overlap set to 1 calendar year (e.g. all of 1970). This avoided the use of default dates but represented a period (from 12 to 23 months) which was both variable and, in the context of young children, lengthy.

Two children were deemed to have had school contact if either they or their siblings had attended the same school for at least one common term. Formal statistical analyses was not appropriate because of the small numbers of children involved.

### *Exploratory analysis*

These have mainly been restricted to examination of subgroups (age, subtype) to determine which cases give rise to the excess numbers of case-to-case pairs, and of the period of linkage for clues as to latency. It is appropriate to think of the targets as being 'susceptible' to some infectious agent during the period of linkage and the NNs as 'infective' – markers, at least, for a source of infection. It may be helpful to suppose that the NNs could shed the agent during the same period but analysis using this methodology cannot provide evidence for this nor for any direct case to case transmission. Contact (direct or indirect) is most likely to have occurred between the beginning and ending of the linkage period. Examination of the results led to a tentative suggestion that some children might be susceptible around the time of birth. To test this, a second analysis was conducted with residential distance defined as before except that for target children the residential history was restricted to the time between conception and the first birthday. We emphasise that this is a '*post-hoc*' analysis but one generated primarily by recursive thought around results rather than data inspection.

All analyses used in-house software.

### Results

Details of the residential-distance pairs are shown in Table I. Approximately half the controls in each area had controls as NN as would be expected but 69 (63%) of cases had cases as NN. This excess was particularly marked for North Humberside. Formal testing of the Cuzick–Edwards statistic $T_1$ (i.e. the number of case → case pairs) showed that the excess case → case linkage was highly significant. The disparity between observed and expected values of $T_k$ decreased rapidly with k and for $T_3$, $T_4$ the ratios of observed to expected values were close to unity. Application of the Bonferroni correction for the four tests yields an overall significant result ($P = 0.024$). The NNs in the 69 case → case pairs included just 51 individual cases with several serving as NN to a number of cases. When the analysis was conducted using a full calendar year of overlap (see methods) the results were in the same direction but considerably weaker; only $T_1$ was significantly elevated. The results reported subsequently all refer to a 6 month overlap.

**Table I** Number of pairs by status, area (Residential – distance: any time from conception to diagnosis

| *Target* | *NN* | *Gateshead* | *Cumbria* | *N. Humbs* | *Total* |
|---|---|---|---|---|---|
| Case | Case | 20 | 16 | 33 | 69 |
| Case | Control | 17 | 9 | 14 | 40 |
| Control | Case | 20 | 12 | 23 | 55 |
| Control | Control | 17 | 13 | 24 | 54 |
| Cuzick–Edwards test† (*P*) | | 0.32 | 0.11 | 0.01 | 0.006 |

†Monte Carlo test based on $T_1$ with 9999 simulations.

Result with targets aged 0–4 years and 5–14 years considered separately (Table II) indicate that the residential proximity to other cases is excessive when the younger cases are taken as targets. Once again these results are most striking for North Humberside. When the analysis of Table II was repeated with the ages of NNs restricted in the same way as for the target children neither group showed evidence of unusual case → case proximity. Inspection of the ages of the NNs revealed that 41 of those in the case → case pairs (i.e. 59%) were aged 5–14 years although only 43 cases (39%) were in that age range. Thus the data display a tendency for younger onset cases to have lived close to older onset cases at some time prior to the diagnosis of either child.

The diagnostic distribution of targets and NNs in case → case pairs (Table III) shows that young ALL cases form a higher percentage of the targets but a lower percentage of the NNs than the overall series. Conversely, older ALL and other diagnoses occur more frequently amongst the NNs. For the 15 target cases whose diagnosis was not ALL a diagnosis of ALL for the NN was not common (6/15 = 40%).

The time period of linkage for case → case and other pairs is related to the dates of birth and diagnosis of the two in Table IV. This table provides some guidance on possible times of infectivity and susceptibility and hence latency. The relationships between linkage and dates of birth are similar for case → case and other pairs but a small excess of target cases with linkage period ending by the date of birth (13% compared with 9%) suggests that a minority of cases may be susceptible prenatally.

Relationships to dates of diagnosis (Table IV) show that both targets and NNs of case → case pairs are somewhat more likely to have the link beginning in the second year prior to diagnosis than other pairs (20% compared with 11%). For five of the case → case pairs the period of linkage was close to both dates of diagnosis. However, the vast majority (over 70%) of case → case pairs had their link extending into periods more than two years prior to the date of diagnosis of at least one child.

Figure 1 illustrates the largest 'cluster' of linked cases. This shows a pattern which is apparent in all three study areas; an older 'pivotal' case is NN to a number a younger (target) cases but the times of linkage show little overlap and are spread over several years. Linkage may also involve different addresses for the pivotal case. The cases in Figure 1 were all ALL and there was a suggestion, here, of excess T cell disease.

Very few children had school contact as defined in Methods (six pairs of cases and five pairs of controls). Manual inspection of the interview forms for the cases in the three largest clusters revealed that each child was linked by common attendance at school and/or pre-school activity (of self or sibling) with at least one other child in the cluster.

Consideration of the results for residential distance led to a *post-hoc* hypothesis that the time around birth might be a 'susceptible' period for some, possibly older, cases. Therefore the Cuzick–Edwards analysis was repeated with residential linkage of target children restricted to the 2 years surrounding birth. There was evidence in all these areas of excess case → case proximity according to the restricted definition (Table V) thus confirming that these data suggest an excess of case children who, around the time of their birth, had lived close to other children who later developed leukaemia. There was no evidence that the target children involved were older than the overall series.

**Table III** Diagnostic groups for targets and NNs in case → case pairs (numbers and column %)

| | *Targets* | *NNs* | *Total study* |
|---|---|---|---|
| ALL (age 0–4 years) | 36 (52%) | 18 (35%) | 47% |
| ALL (age 5–14 years) | 18 (26%) | 18 (35%) | 29% |
| AML | 5 ( 7%) | 5 (10%) | 6% |
| Other leukaemia | 5 ( 7%) | 4 ( 8%) | 5% |
| NHL | 5 ( 7%) | 6 (12%) | 13% |
| Total | 69 | 51 ) | 109 |

ALL = acute lymphoblastic leukaemia; AML = acute myeloid leukaemia; NHL = non-Hodkin's lymphoma.

### Discussion

The study used case-control methodology to investigate a retrospective population-based series of childhood leukaemia and NHL cases in three geographical locations. Gateshead is entirely urban and of predominately lower socioeconomic status; the other areas are of relatively higher status and largely rural, although North Humberside contains the city of Kingston-upon-Hull. The study was restricted to 'eligible' case children who were both born and diagnosed in the same study area and contributed 71.1% for all cases ascertained. The distribution of diagnostic subgroups was similar in both the eligible and ineligible case series with ALL being diagnosed in 78% and 67% respectively. Each geographical area had a similar proportion of eligible cases. However, the two groups had different age structures with 0–4 year olds comprising 61% of the eligible children and 35% of the ineligible group. This differential is not surprising as older children (5–14 years) are more likely to have moved away from their place of birth thereby making them ineligible.

The results contribute to the body of literature on clustering and social contact of leukaemia cases and require interpretation in this context. Numerous anecdotal reports of clusters of leukaemia especially among children can be found in the literature. One of the most striking occurred in Niles, Illinois (Heath & Hasterlick, 1963). These reports, are uninterpretable because of the absence of formal statistical analysis.

A variety of formal methodologists have been applied (Linet, 1985). Most common are tests of space-time clustering (e.g. Knox, 1964); controls are not required but attention must focus on one particular date (usually date of birth or date of diagnosis). Conflicting reports (reviewed in Linet, 1985) include both weak positive and negative results. A small number of studies have investigated spatial clustering of childhood leukaemias (Cartwright *et al.*, 1988; Openshaw *et al.*, 1988; Lewis, 1980; Comare, 1988). Methodologies

**Table II** Number of pairs by age of target, status and area (residential – distance: any time from conception to diagnosis.)

| | *Status* | | *Area* | | | |
|---|---|---|---|---|---|---|
| *Age of Target* | *Target* | *NN* | *Gateshead* | *Cumbria* | *N. Humbs* | *Total* |
| 0–4 years | Case | Case | 13 | 10 | 22 | 45 |
| | Case | Control | 9 | 7 | 15 | 21 |
| Cuzick–Edwards test* (*P*) | | | 0.22 | 0.19 | 0.001 | 0.002 |
| 5–14 years | Case | Case | 7 | 6 | 11 | 24 |
| | Case | Control | 8 | 4 | 8 | 19 |
| Cuzick–Edwards test* (*P*) | | | 0.60 | 0.20 | 0.25 | 0.23 |

*Monte Carlo test with 9999 simulations; Results are only shown for pairs with cases as targets.

**Table IV** Relationship of period of linkage to other key dates by status of pair (numbers and column percentages)

| | | Status of pair Case → case | Other |
|---|---|---|---|
| *Relationship to dob of target* | | | |
| Link began | before dob | 34 (49.3%) | 77 (51.7%) |
| | 1st year of life | 7 (10.1%) | 18 (12.1%) |
| | Later | 28 (40.6%) | 54 (36.2%) |
| Link ended | at/before dob | 9 (13.0%) | 14 ( 9.4%) |
| | 1st year of life | 8 (11.5%) | 17 (11.4%) |
| | later | 52 (75.4%) | 118 (79.2%) |
| *Relationship to dob of NN* | | | |
| Link began | before dob | 28 (40.6%) | 71 (47.0%) |
| | 1st year of life | 8 (11.5%) | 21 (14.1%) |
| | Later | 33 (47.8%) | 57 (38.3%) |
| Link ended | at/before dob | 6 ( 8.7%) | 12 ( 8.1%) |
| | 1st year of life | 9 (13.8%) | 18 (12.1%) |
| | Later | 54 (78.3%) | 119 (79.9%) |
| *Relationship to both dob's* | | | |
| Link began | before/around dobs | 4 ( 5.7%) | 14 ( 9.4%) |
| | Later | 65 (94.2%) | 135 (90.6%) |
| *Relationship to diagnosis date of target* | | | |
| Link ended | at diagnosis | 36 (52.3%) | 78 (52.3%) |
| | 1st year before diag | 9 (13.0%) | 15 (10.1%) |
| | Earlier | 24 (34.7%) | 56 (37.6%) |
| Link began | 1st year before diag | 2 ( 2.9%) | 12 ( 8.1%) |
| | 2nd year before diag | 14 (20.3%) | 17 (11.4%) |
| | Earlier | 53 (76.8%) | 120 (80.5%) |
| *Relationship to diagnosis date of NN* | | | |
| Link ended | at diagnosis | 32 (46.4%) | 58 (38.9%) |
| | 1st year before diagnosis | 7 (10.1%) | 15 (10.1%) |
| | Earlier | 30 (43.5%) | 76 (51.0%) |
| Link began | 1st year before diag | 3 (4.3%) | 10 ( 6.7%) |
| | 2nd year before diag | 14 (20.3%) | 15 (10.1%) |
| | Earlier | 52 (75.4%) | 124 (83.2%) |
| *Relationship to both dates of diagnoses* | | | |
| Link ended | at/around diagnoses | 5 ( 7.2%) | 5 ( 3.3%) |
| | 1st year before both diagnosis | 9 (13.0%) | 19 (12.8%) |
| | Earlier | 55 (79.7%) | 125 (83.9%) |

dob = date of birth.

**Figure 1** Target → neighbour linkages (→) for one group of cases. Ages are ages at diagnosis. If the year of birth of case X is taken as year 0 then: A→X linkage was year 2–year 3; B→X linkage was year 0–year 1; C→X linkage was year 5–year 8; D→X linkage was year -1–year 1; E→D linkage was year 6–year 8. Linkages for case X involved three district addresses. Diagnoses are: A, T cell ALL; B, non-T, non-B-ALL; C, ALL not classified; D, T cell ALL; E, common ALL; X, common ALL.

differ, but, again, events must be located once, usually at diagnosis. Recent independent analyses of a large UK national data set have shown convincing evidence of weak spatial clustering of ALL cases diagnosed over lengthy time periods (OPCS, 1991).

Pike & Smith (1974) used lifetime residential histories to quantify the possibility of 'effective' contact during postulated periods of 'infectivity' and 'susceptibility'. An application of this method to childhood leukaemias (Smith *et al.*, 1976) applied a large number of tests for different definitions of each of the three variables; one of the most significant results fixed susceptibility around birth but infectivity any time from birth to diagnosis.

The strength of the present approach is that the data determine the times at which linkage is important and no prior hypotheses are required. We have found a significant excess of cases having other cases living nearby (i.e. nearest neighbours) for periods of 6 months or more. This does not extend either to more distant neighbours or to lengthy periods of residential overlap. There is, in consequence, some ambiguity but detailed examination of the nearest neighbour pattern permits a consistent interpretation. Firstly there is evidence for linkage of groups of 'target' cases around a smaller number of pivotal 'neighbour' cases of which Figure 1 is the most striking example. That the diagnoses were all ALL provides some biological plausability for the inference of an aetiological link. Secondly, the target cases tend to be younger onset ALL and residential proximity need only have persisted for a few months.

Long recall periods and, consequently, approximate dates of removal are unavoidable features of this study, which may possibly have led to artefactual results; there was, however, no evidence that accuracy of recall for case and control parents differed. It is conceivable, too, that the results are merely manifestations of the localised spatial clustering which

**Table V** Number of pairs by status, area (restricted residential distance: target residences-conception to first birthday, NN residences-conception to diagnosis)

| *Status* | | *Area* | | | |
|---|---|---|---|---|---|
| *target* | *NN* | *Gateshead* | *Cumbria* | *N. Humbs* | *Total* |
| Case | Case | 19 | 14 | 28 | 61 |
| Case | Control | 16 | 8 | 15 | 39 |
| Cuzick–Edwards test* (*P*) | | 0.09 | 0.09 | 0.08 | 0.002 |

*Monte Carlo test based on $T_1$ with 9999 simulations; Results are only shown for pairs with cases as targets.

had already been identified in these areas (Craft & Birch, 1983; Cartwright *et al.*, 1988; Openshaw *et al.*, 1988). This is unlikely since application of the same methods to the present data to test for spatial clustering by location at diagnosis gave significant results only for Gateshead. In addition, the case → case pairs in North Humberside did not include any cases from the post-code sector, HU10, to which the original reports of clustering applied.

The Cuzick–Edwards methodology is a valid approach to investigating spatial clustering when the underlying population is heterogeneous so that longer distances should be regarded as 'close' in rural than in urban areas (Cuzick & Edwards, 1990). It application in the present context is new and must be regarded as somewhat exploratory; it seemed possible that distinct migration patterns might explain the results but, apart from the gestation period, there were no significant case-control differences in migration (Alexander *et al.*, unpublished results). For use of a matched design with the Cuzick–Edwards method matching criteria will be important. The neighbourhood matching we have used is similar to that recommended by Pike & Smith (1974).

No excess case contact at school was observed. The school/pre-school linkages of cases within the three largest clusters shows that social contact could have taken place but the data are essentially anecdotal since they cannot be compared with comparable control information. We have no evidence that personal contact ever occurred.

For all these reasons a cautious interpretation is required. We emphasise that the possibility of direct case to case transmission of any infective agent *cannot* be inferred from these data.

A variety of models for components of infectious aetiologies of childhood leukaemia have been considered. These include case-to-case transmission of a rare infectious agent, an unusual host response to a common infection, abnormal exposure under circumstances of disregulated herd immunity, gestational exposure of the mother (Gilman *et al.*, 1989; Anonymous, 1990; Kinlen *et al.*, 1990; Fleming, 1991). In each of these, the involvement of a specific agent or group of agents is proposed. In addition, Greaves' hypothesis (1988) suggests that protection from general infections and hence absence of antigenic challenge during infancy may be a cause of leukaemia. These are not mutually exclusive and could be combined in multifactorial aetiologies. For example, early isolation from general infection might be followed by an unusual host response to a specific agent (Greaves & Alexander, unpublished).

To interpret the present results we may consider the target cases to be 'susceptible' to infection and the NN cases to be 'infective'. The nearest neighbour pattern suggests that a minority of (older-onset) ALL cases may be 'infective' over lengthy periods during the time from conception to diagnoses.

Most of the susceptible cases are ALL diagnoses at the childhood peak ages and susceptibility may be focused in the two years before the onset of disease. Transmission through community micro-epidemics appears probable but we note that the data are consistent with other common source exposures localised in time and space.

The results suggest the following tentative hypothesis. Some specific agent (Z) can contribute to the aetiology of childhood leukaemia in two distinct ways: (i) in children exposed to Z *in utero* or very early life persistent infection-may be established with consequent risk of developing ALL, primarily beyond the 'childhood peak' years, (ii) postnatal exposure to Z may contribute to the development of ALL at younger ages and, in particular, (iii) ALL in the 'childhood peak' (age 2–4 years) may be rare consequence of the antigenic challenge following relatively late first exposure to Z (and perhaps other infections). Model (i) is related to the putative childhood leukaemia virus discussed in a recent editorial (Anonymous, 1990) and is not supported by epidemiological evidence for the generality of cases – at least in countries with the typical age distribution of developed societies (pattern III of Fleming, 1991). Under Greaves' hypothesis (1988) early isolation (Alexander *et al.*, 1990) and protection from infections in these societies could provide host circumstances favourable to common ALL in the childhood peak under model (iii). Models (ii) and (iii) also relate closely to the work of Kinlen *et al.* (1990) whose results suggest that high doses of some specific infectious agent(s) may be causative aetiological factors for leukaemia in this age group. The hypothesis is consistent with the anecdotal cluster reports and the weak/ambiguous results of other tests of clustering both of which have been discussed earlier.

A testable prediction of the hypothesis was that cases should have lived, at birth, close to children who would later develop leukaemia. This has been confirmed in the present data (Table V), in the study of Smith & Pike (1976) and in a further study (Alexander, 1992) motivated by the present results. The ages of the children are not entirely consistent but this will be, in part, attributable to the study eligibility criteria.

In conclusion, significant clustering of cases of childhood leukaemia by residential proximity has been found, and this has led to generation of specific hypotheses. Confirmation of the results with larger data sets, more recent and more precise data are required. This precision should include both exact dates of removal and immunophenotyping of ALLs neither of which were available here.

The Leukaemia Research Fund support the Clinical Epidemiology Centre at Leeds University. We wish to thank the following medical collaborators on the study: Dr C.C. Bailey, Dr I. Lewis (Leeds), Dr I. Beddis, Dr A.V. Sheard, Dr J.M. Dunlop (Hull), Dr A.W. Craft, Dr J. Kernahan (Newcastle), Dr D.F. Henley (Gateshead), Dr J. Platt, Dr M.B.R. Roberts, Dr J. Terrell, Dr J. Munro, Dr M. Jepson, Dr N. West (Cumbria), Dr P. Morris-Jones, Dr R. Stevens (Manchester). The following interviewers are acknowledged for their assiduous data collection: P. Roberts, J. O'Sullivan (Leeds), B. Routledge, A. Ulyett, C. Wilson (Cumbria), D. Fadden, M. Moore (Gateshead). We are grateful to L. Parker and J. Hargreaves for administration, developement and support work, to B. Pearlman for interviewer training and Y. Gibbons and S. Fizpatrick for coding. We also thank the FPC and District Health Authority staff who facilitated our work. J. Williams, J. Carrette and D. Rowland are thanked for help with the computing and A. Pickles, A. McKeating and J. Pedder for typing the manuscript. H. Lilley and the Yorkshire Children's Tumour Registry, supported by the Leeds Candlelighters, are thanked for continued assistance. The collaboration of L. More from the Northern Children's Malignant Disease Registry is gratefully acknowledge.

Dr Gerald Draper and the anonymous referees are thanked for their comments on the first draft of this paper.

### References

ALEXANDER, F.E (1992). Space-time clustering of childhood-leukaemia: indirect evidence for a transmissible agent. *Br. J. Cancer*, **65**, 589-592.

ALEXANDER, F.E., MCKINNEY, P.A., RICKETTS, T.J. & CARTWRIGHT, R.A. (1990a). Community lifestyle characteristics and risk of acute lymphoblastic leukaemia in children. *Lancet*, **336**, 1461.

ALEXANDER, F.E., MCKINNEY, P.A., CARTWRIGHT, R.A. & RICKETTS, T.J. (1990b). Investigation of spatial clustering of rare diseases: childhood malignancies in North Humberside. *J. Epidemiol. Community Health* **44**, 39.

ANONYMOUS (1990). Childhood leukaemia: an infectious disease? (Editorial) *Lancet*, **336**, 1477.

BAXTER, M.S., EAST, B.W., MACKENZIE, A.B. & SCOTT, E.M. (1990). A review of radioactivity in and around the Capper Pass Smelter, Melton Works, North Humberside. Scottish Universities Research and Reactor Centre Report prepared for East Yorkshire Health Authority.

BLACK, D. (1984). Investigation of the possible increased incidence of cancer in West Cumbria. Report of Independent Advisory Group. HMSO: London.

CARTWRIGHT, R.A., ALEXANDER, F.E. & MCKINNEY, P.A. (1988). Report on childhood cancer aggregations in the Beverley and Kingston-upon-Hull administrative districts. Report submitted to the East Yorkshire Health Authority.

COMMITTEE ON MEDICAL ASPECTS OF RADIATION IN THE ENVIRONMENT (COMARE) (1988). Second report. Investigation of the possible increased incidence of leukaemia in young people near the Dounrey Nuclear Establishment, Caithness, Scotland. HMSO: London.

CRAFT, A.W. & BIRCH, J.M. (1983). Childhood cancer in Cumbria. *Lancet*, **ii**, 1299.

CUZICK, J. & EDWARDS, R. (1990). Tests for spatial clustering in heterogeneous populations. *J. Roy Statis. Soc. Series B*, **52**, 73.

DARBY, S.C. & DOLL, R. (1987). Fallout radiation doses near Dounreay and childhood leukaemia. *Br. Med. J.*, **294**, 603.

FLEMING, A.F. (1991). Childhood leukaemia. Letter. *Lancet*, **337**, 361.

GARDNER, M.J., HALL, A.J., DOWNES, S. & TERRELL, J.D. (1987a). Follow-up study of children born to mothers resident in Seascale, West Cumbria (Birth Cohort). *Br. Med. J.*, **295**, 822.

GARDNER, M.J., HALL, A.J., DOWNES, S. & TERRELL, J.D. (1987b). Follow up study of children born elsewhere but attending schools in Seascale, West Cumbria. (Schools cohort). *Br. Med. J.*, **295**, 819.

GARDNER, M.J., SNEE, M.P., HALL, A.J., POWELL, C.A., DOWNES, S. & TERRELL, J.D. (1990). Results of case-control study of leukaemia and lymphoma among young people near Sellafield nuclear plant in West Cumbria. *Br. Med. J.*, **300**, 423.

GILMAN, E.A. & others (1989). Childhood cancers and their association with pregnancy drugs and illnesses. *Paediatr. Perinatal Epidemiol.*, **3**, 66.

GREAVES, M.F. (1988). Speculations on the cause of childhood acute lymphoblastic leukaemia. *Leukaemia*, **2**, 120.

HEATH, C.W. & HASTERLICK, R.J. (1963). Leukaemia among children in a suburban community. *Am. J. Med.*, **34**, 796.

KINLEN, L. (1988). Evidence for an infective cause of childhood leukaemia comparison of a Scottish New Town with nuclear reprocessing sites in Britain. *Lancet*, **II**, 1323.

KINLEN, L., CLARK, K. & HUDSON, C. (1990). Evidence from population mixing in British New Towns 1946-85 of an infective basis for childhood leukaemia. *Lancet*, **336**, 577.

KNOX, E.G. (1964). The detection of space-time interactions. *Appl. Statist.*, **13**, 25.

LEWIS, M.S. (1980). Spatial clustering in childhood leukaemia. *J. Chron. Dis.*; **33**, 703.

LINET, M.S. (1985). *The Leukaemias: Epidemiological Aspects. Monographs in Epidemiology and Biostatistics.* Oxford University Press: New York.

MCKINNEY, P.A., ALEXANDER, F.E., CARTWRIGHT, R.A. & PARKER, L. (1991). The parental occupations of children with leukaemia in West Cumbria, North Humberside and Gateshead. *Br. Med. J.*, **302**, 681.

OPCS (1991). *The Geographical Epidemiology of Childhood Leukaemia and non-Hodgkin lymphoma in Great Britain 1966-83*, Draper, G. (ed.). HMSO: London.

OPENSHAW, S., CRAFT, A.W., CHARLTON, M. & BIRCH, J.M. (1988). Investigation of leukaemia clusters by use of a geographical analysis machine. *Lancet*, **i**, 272.

PIKE, M.C. & SMITH, P.G. (1974). A case-control approach to examine diseases with for evidence of contagion, including diseases with long latent periods. *Biometrics*, **30**, 263.

SMITH, P.G., PIKE, M.C., TILL, M.M. & HARDISTY, R.M. (1976). Epidemiology of childhood in Greater London: A search for evidence of transmission assuming a possibly long latent period. *Br. J. Cancer*, **33**, 1.